# Genetic and epigenetic determinants of vitamin D metabolism: nutrigenomic insights for precision nutrition

**DOI:** 10.3389/fnut.2026.1772849

**Published:** 2026-03-06

**Authors:** Fatima Qahtan, Salma Abu-Qiyas, Dimitrios Papandreou

**Affiliations:** Department of Clinical Nutrition and Dietetics, College of Health Sciences, University of Sharjah, Sharjah, United Arab Emirates

**Keywords:** epigenetics, nutrigenetics, nutrigenomics, precision nutrition, vitamin D

## Abstract

Vitamin D plays a pivotal role in immune regulation, metabolic balance, skeletal health, and gene expression. Growing evidence indicates that genetic and epigenetic factors contribute to interindividual differences in vitamin D status and physiological responses. This review summarizes current findings on the nutrigenomic determinants of vitamin D metabolism, with emphasis on genetic polymorphisms in vitamin D receptor (VDR), GC, CYP2R1, CYP27B1, and CYP24A1, as well as epigenetic mechanisms that modulate vitamin D related gene expression. Peer-reviewed original studies and review articles published between 2010 and 2025 were examined to highlight associations between genetic variation in the vitamin D pathway and susceptibility to cancer, autoimmune disorders, metabolic diseases, cardiovascular conditions, and neurodegenerative outcomes. Advances in omics technologies and epigenetic biomarker research have improved understanding the molecular pathways through which vitamin D acts across multiple body systems. Evidence from gene–environment interactions and genotype-specific supplementation responses highlights the conceptual relevance of precision nutrition, while underscoring substantial gaps in clinical validation. Collectively, current research suggests that genetic information may inform future personalized vitamin D strategies, although translation into clinical practice remains limited by inconsistent evidence and methodological heterogeneity.

## Introduction

Vitamin D is one of the most extensively studied micronutrients in modern nutritional science, particularly because of its wide-ranging effects on immune regulation, metabolic function, bone health, and gene expression. Through its nuclear receptor, vitamin D influences the activity of hundreds of genes, underscoring its broad physiological significance. In recent years, nutrigenomics the study of interactions between nutrients and the genome has provided valuable insight into why individuals vary widely in their response to vitamin D intake and supplementation (1). This emerging area highlights how genetic differences shape vitamin D absorption, metabolism, transport, and cellular action, ultimately influencing disease risk and health outcomes.

Vitamin D deficiency remains a major global health issue, affecting nearly half of the world’s population and contributing to elevated risks of chronic diseases (2). Although sunlight exposure and dietary intake are major determinants of vitamin D status, they do not fully explain the considerable variability observed among individuals. Genetic factors play a significant role in influencing circulating vitamin D concentrations, and these genetic differences help explain why some individuals remain deficient even when following standard supplementation guidelines. Identifying these genetic determinants is therefore important for understanding interindividual variability in vitamin D status and for informing future research in precision nutrition (3).

In this context, this review synthesizes selected evidence on the genetic, epigenetic, and molecular determinants of vitamin D metabolism, with the aim of critically evaluating the strength, consistency, and limitations of current nutrigenomic findings and clarifying their implications and constraints for personalized nutrition and disease prevention.

Despite growing research on vitamin D genetics, most studies focus on isolated genes or single polymorphisms and do not integrate findings across the entire vitamin D metabolic pathway. This limit understanding of how multiple genetic, epigenetic, and nutrigenomic factors work together to influence vitamin D status and disease risk. Evidence linking genetic and epigenetic variation to vitamin D status and health outcomes arises from diverse study designs with differing levels of inferential strength. Genome-wide association studies identify robust population-level signals but generally explain only a small proportion of interindividual variability. Candidate-gene and case–control studies often report larger effects but show greater heterogeneity and limited reproducibility. Epigenetic studies are predominantly cross-sectional and associative, while mechanistic experiments provide biological plausibility but limited direct clinical translatability. These differences are explicitly considered throughout this review when interpreting reported associations.

This review integrates evidence across vitamin D synthesis, transport, metabolism, and receptor signaling to provide a pathway-level overview that brings together findings previously examined in isolation. In addition, across genetic, epigenetic, and molecular domains, this review distinguishes relatively robust population-level associations from more speculative or context-dependent mechanisms, particularly where evidence is associative, population specific, or derived from experimental models.

## Methodology

To strengthen the analytical rigor of this review, the available evidence was evaluated using a clear qualitative hierarchy. Results from replicated genome-wide association studies and Mendelian randomization analyses were treated as the most robust sources of population-level evidence. In contrast, findings from candidate-gene studies were interpreted with greater caution, acknowledging their context- and population-specific nature, particularly in cases of limited or inconsistent replication. Epigenetic and mechanistic studies were primarily used to support biological plausibility rather than to infer causality. This structured approach informed the weighting and interpretation of the pathways and outcomes discussed in the following sections. This narrative review followed the PRISMA-S extension for transparent reporting of literature searches and the SANRA 2.0 (Scale for the Assessment of Narrative Review Articles) recommendations for high-quality narrative reviews, as applicable Although this review is narrative in scope and does not follow a formal systematic review or meta-analytic framework, elements of PRISMA-S were applied to enhance transparency and reproducibility of the literature search and study selection process, consistent with best-practice recommendations for narrative reviews. The literature search was structured around predefined conceptual blocks capturing vitamin D biology, genetic and epigenetic regulation, and applications in nutrigenomics and precision nutrition. Concept blocks were combined using Boolean operators, with “AND” applied between conceptual domains and “OR” applied within domains. Controlled vocabulary [Medical Subject Headings (MeSH) in PubMed] and free-text terms were adapted as appropriate for each database (PubMed, Scopus, and Google Scholar), including the use of truncation and variant spellings. In addition, the reference lists of all included reviews and eligible primary studies were manually screened to identify further relevant publications. The primary exposure/pathway block included terms related to vitamin D and its signaling components, such as “vitamin D,” “cholecalciferol,” “ergocalciferol,” “25-hydroxyvitamin D,” “calcitriol,” “vitamin D receptor,” and “VDR.” The genetic and epigenetic block included terms reflecting variation and regulatory mechanisms, including “genetic polymorphism,” “single nucleotide polymorphism (SNP),” “genome-wide association study (GWAS),” “epigenetic,” “DNA methylation,” and “histone modification.” The nutrigenomics and precision nutrition block included terms such as “nutrigenomics,” “nutrigenetics,” “precision nutrition,” and “personalized nutrition.” Search strategies were iteratively refined to improve specificity while preserving conceptual scope and were adapted to the syntax and indexing practices of each database. Final searches were limited to human studies published in English between January 2010 and January 2025.

**Figure 1:**
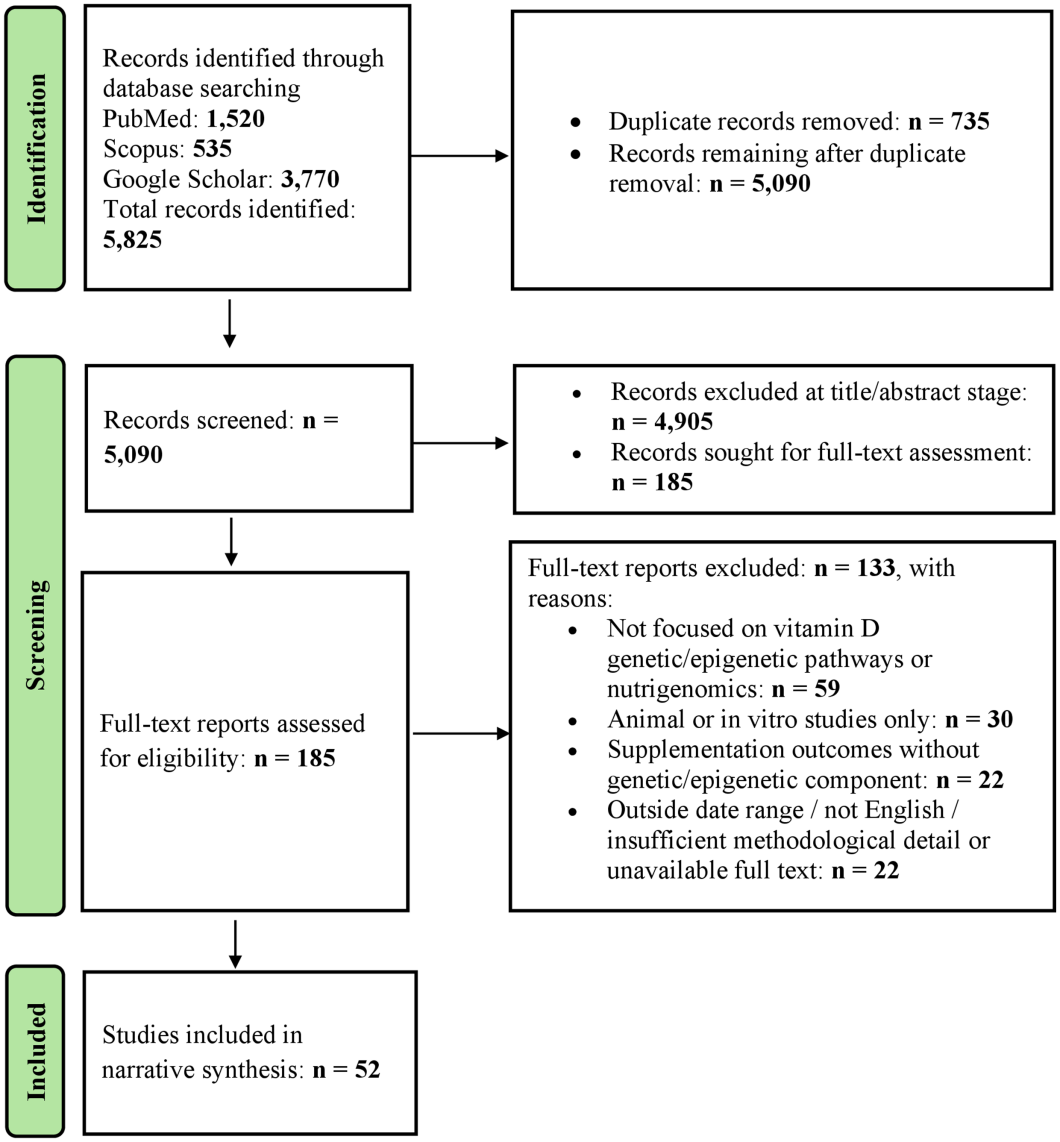
PRISMA-style flow diagram of literature identification and study selection.

Study identification and selection were guided by conceptual relevance with predefined criteria to ensure focused inclusion of literature addressing vitamin D metabolism, genetic and epigenetic regulation, as well as nutrigenomics in diverse study designs. Studies were included if they met the following criteria: (1) publication between January 2010 and January 2025; (2) English-language publication; (3) involvement of human participants; (4) examination of vitamin D status, metabolism, or signaling, including the vitamin D receptor (VDR); (5) investigation of genetic polymorphisms, epigenetic mechanisms, or gene–nutrient interactions relevant to vitamin D; and (6) relevance to nutrigenomics, epigenetics, or precision and personalized nutrition. Both original research articles (including observational studies, randomized controlled trials, and genetic association studies) and narrative or systematic review articles were considered. Studies were excluded if they (1) were conducted exclusively in animal or *in vitro* models; (2) did not specifically address vitamin D-related genetic, epigenetic, or metabolic pathways; (3) focused solely on clinical supplementation outcomes without a genetic, epigenetic, or precision nutrition component; (4) were conference abstracts, editorials, commentaries, case reports, or theses; or (5) lacked sufficient methodological detail or full-text availability. Articles not published in English were also excluded. The narrative synthesis combines evidence from observational studies, genetic association studies, clinical investigations, and relevant reviews to provide an overview of vitamin D metabolism and regulation. A PRISMA-style flow diagram was constructed to visually summarize the literature identification, screening, eligibility assessment, and inclusion process (Figure 1).

All records retrieved from database searches were imported into a reference management software, where duplicate citations were identified and removed using automated tools followed by manual verification. A total of 735 duplicate records were removed, leaving 5,090 unique records for screening. Title and abstract screening was performed independently by two reviewers according to predefined inclusion and exclusion criteria. Disagreements were resolved by discussion, with consultation from a third reviewer when required.

Of the 5,090 records screened, 4,905 were excluded at the title and abstract stage. 185 articles were retrieved for full-text assessment. Full-text articles were independently reviewed by the same reviewers, resulting in the exclusion of 133 studies for the following reasons: lack of focus on vitamin D genetic or epigenetic pathways or nutrigenomics (*n* = 59), animal or *in vitro*-only studies (*n* = 30), supplementation studies without a genetic or epigenetic component (*n* = 22), and articles outside the specified date range, non-English publications, or insufficient methodological detail or unavailable full text (*n* = 22). Ultimately, 52 studies were included in the final narrative synthesis.

### Biosynthesis and metabolic pathways

**Figure 2:**
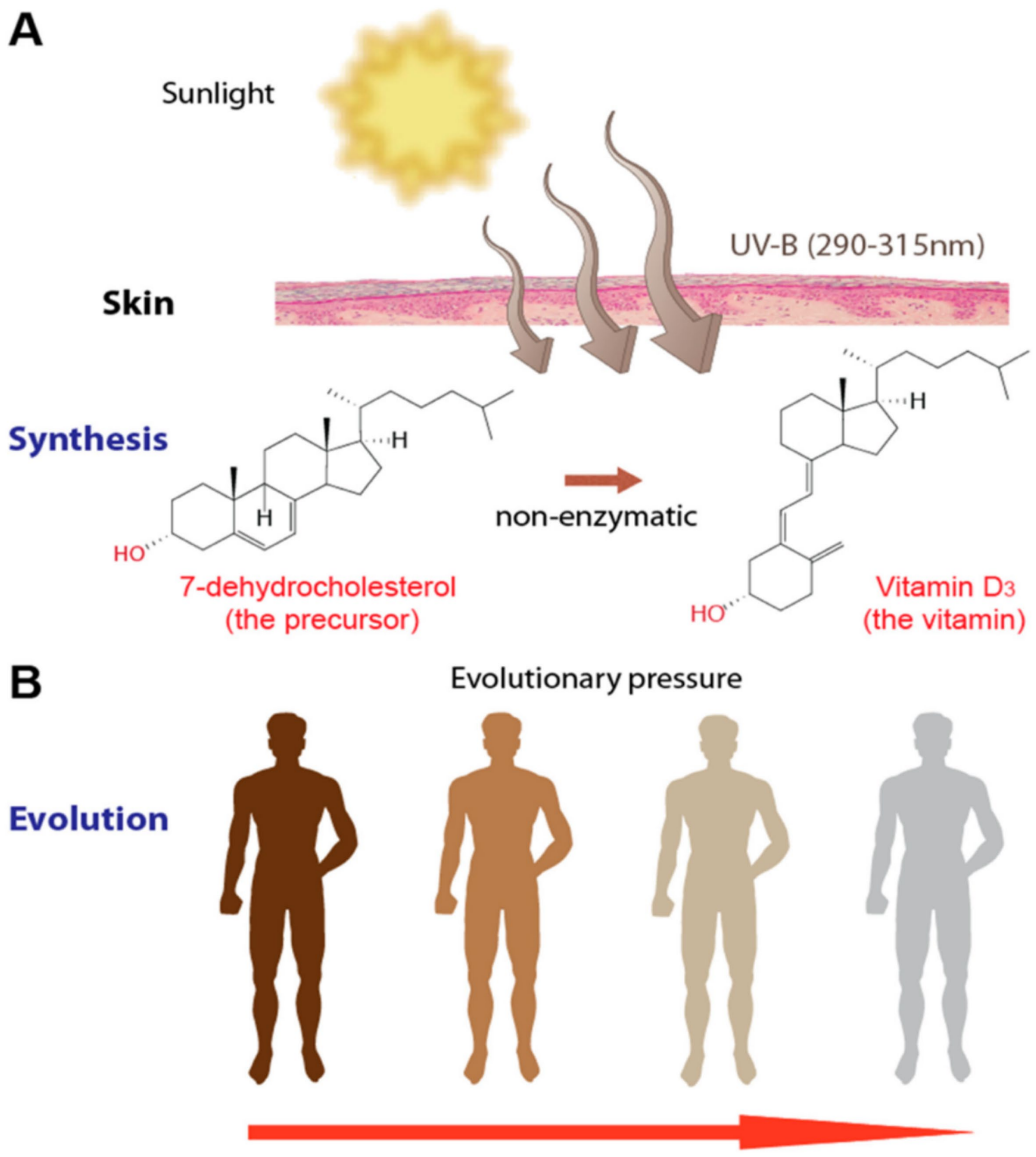
Vitamin D₃ synthesis and evolutionary adaptation. Adapted from Carlberg (2), licensed under CC BY 4.0. **(A)** UV-B radiation (290–315 nm) converts 7-dehydrocholesterol in the skin into vitamin D₃ through a non-enzymatic reaction. **(B)** Evolutionary differences in skin pigmentation reflect adaptations to varying UV exposure, influencing vitamin D synthesis efficiency.

Vitamin D exists in two primary forms, ergocalciferol (D₂) and cholecalciferol (D₃), both of which must undergo two hydroxylation steps to become biologically active (1). Endogenous synthesis begins in the skin, where ultraviolet-B radiation converts 7-dehydrocholesterol into previtamin D₃ before thermal isomerization produces vitamin D₃. This process is summarized visually in Figure 2, which shows the UVB-mediated conversion of 7-dehydrocholesterol to vitamin D₃. Together with dietary intake, this contributes to circulating vitamin D levels, which are transported in the bloodstream bound to vitamin D-binding protein. Once absorbed, vitamin D undergoes its first hydroxylation in the liver by 25-hydroxylase enzymes, primarily CYP2R1, forming 25-hydroxyvitamin D (calcidiol), the major circulating form used to assess vitamin D status (4). The second hydroxylation occurs in the kidneys through the enzyme 1α-hydroxylase (CYP27B1), producing calcitriol, the biologically active hormonal form. Vitamin D metabolites are inactivated through 24-hydroxylase (CYP24A1), which regulates their breakdown (5). These metabolic pathways are tightly controlled and influenced by significant genetic variation (1).

### Genes related to vitamin D metabolism

Several genes play essential roles in vitamin D transport, activation, and cellular signaling, making them central to nutrigenomic research. The vitamin D receptor (VDR) gene encodes the nuclear receptor responsible for mediating most biological actions of vitamin D through binding to vitamin D response elements in target genes (6). Common VDR polymorphisms including FokI (rs2228570), BsmI (rs1544410), ApaI (rs7975232), TaqI (rs731236), and Cdx2 (rs11568820), have been widely investigated for associations with vitamin D status and disease susceptibility (4).

Another key determinant of vitamin D physiology is the GC gene, which encodes vitamin D- binding protein. Variants such as the widely studied rs2282679 polymorphism have been consistently linked to lower serum vitamin D levels across diverse populations (4). However, circulating 25-hydroxyvitamin D concentrations do not necessarily reflect tissue-level vitamin D bioavailability or downstream signaling activity, particularly in the presence of genetic variation affecting transport or receptor function (4). Because vitamin D-binding protein is responsible for vitamin D transport and bioavailability, variants in GC significantly influence circulating vitamin D levels independent of intake.

Genetic variants in CYP2R1 (such as rs10741657) affect 25-hydroxylase activity, altering the efficiency of conversion from vitamin D to its major circulating form (3). Similarly, polymorphisms in CYP27B1 and CYP24A1 influence the activation and inactivation of vitamin D metabolites (4). Large genome-wide association studies have repeatedly identified CYP2R1 as a major genetic determinant of vitamin D status (7).

#### VDR polymorphisms

VDR polymorphisms are among the most widely studied genetic determinants of vitamin D responsiveness, although their individual effect sizes are generally modest. The FokI variant alters the start codon of the VDR gene, producing receptor isoforms with different transcriptional activity. The shorter isoform has been reported to exhibit greater transcriptional activity, which may contribute to enhanced vitamin D signaling and variable supplementation responses in some populations (8). In addition to coding-region variants, promoter polymorphisms such as Cdx2 also influence VDR function. The Cdx2 (rs11568820) polymorphism is a promoter-region variant located upstream of exon 1 that decreases VDR gene promoter activity, potentially reducing VDR expression (6).

**Figure 3:**
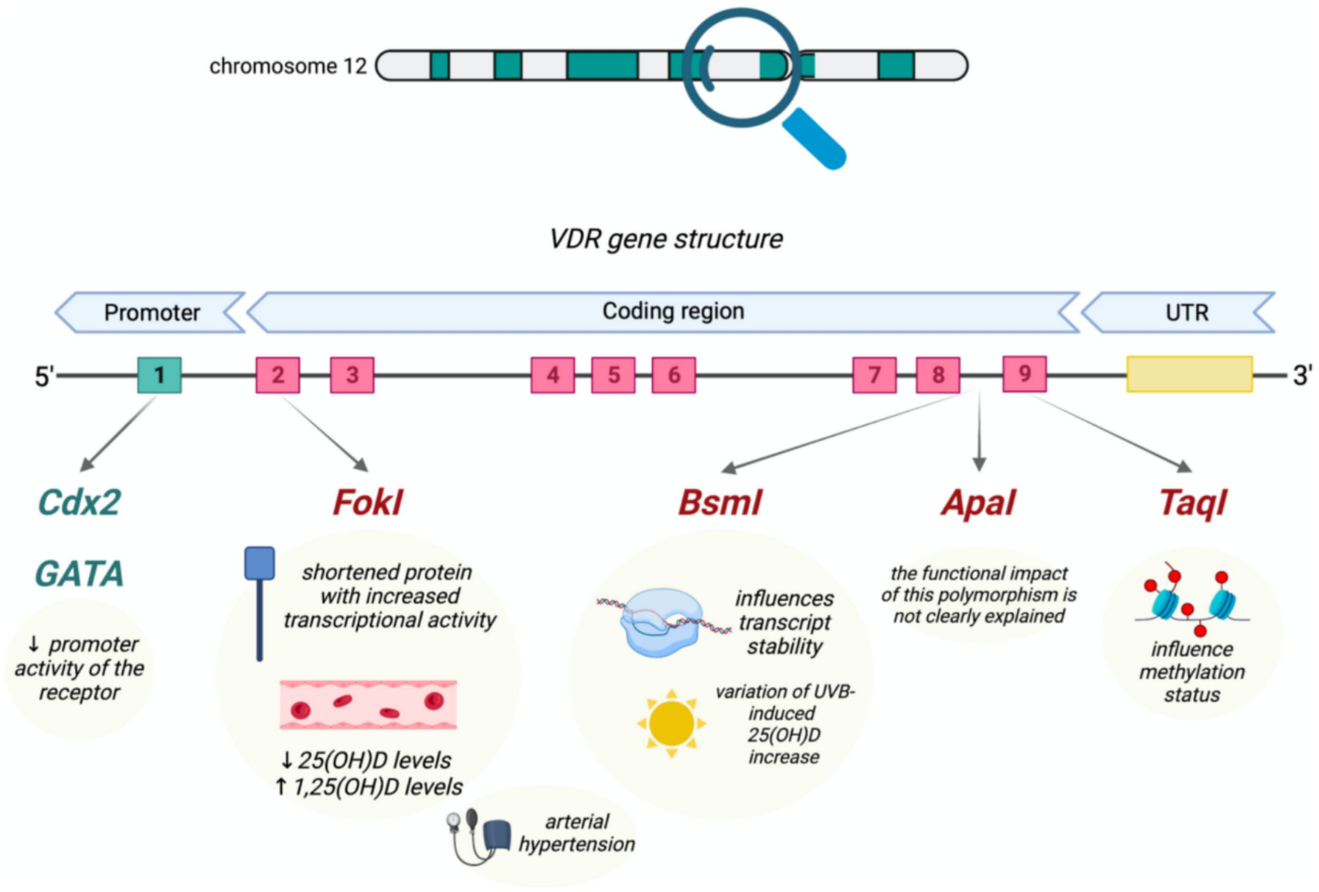
Structure of the vitamin D receptor (VDR) gene and major polymorphisms. Adapted from Voltan et al. (6), licensed under CC BY 4.0. Common SNPs-Cdx2, FokI, BsmI, ApaI, and TaqI-occur in promoter, coding, and UTR regions and influence transcriptional activity, mRNA stability, methylation patterns, and serum vitamin D levels.

Other common VDR variants (BsmI, ApaI, and TaqI) are located in the 3′ (UTR) and are often inherited together as a haplotype block. As a result, these polymorphisms may act as markers in linkage disequilibrium with functional variants rather than exerting direct biological effects themselves. These polymorphisms have been associated with circulating vitamin D levels as well as disease susceptibility, including outcomes in hepatocellular carcinoma and breast cancer risk (9). Figure 3 illustrates the genomic structure of the VDR gene and the location of major polymorphisms, along with their reported functional effects.

Several polymorphisms in the vitamin D receptor (VDR) gene have been shown to influence cellular responsiveness to vitamin D rather than circulating 25-hydroxyvitamin D concentrations, with effects that are generally modest and population dependent. The rs10783219 variant has been associated with lower circulating 25-hydroxyvitamin D concentrations and an increased risk of vitamin D deficiency (8, 10, 11). In contrast, associations for rs1544410 (BsmI) have been inconsistent across populations, with some studies reporting positive associations with serum vitamin D levels and others observing no direct relationship (10, 12, 13). Such inconsistencies underscore the limited predictive value of individual VDR polymorphisms and highlight the influence of ancestry, baseline vitamin D status, and environmental context. These inconsistencies likely arise from differences in allele frequencies, study design, baseline vitamin D status, and interactions with environmental factors such as sunlight exposure and dietary intake. Notably, rs1544410 may influence vitamin D metabolism indirectly by altering *VDR* expression and regulating downstream genes such as *CYP24A1*, thereby affecting vitamin D metabolism without directly changing circulating levels (14). The rs2228570 (FokI) polymorphism has also been linked to variability in vitamin D status and supplementation response, with genotype-specific differences reported across populations (8, 10). Overall, these findings suggest that *VDR* variants primarily modulate vitamin D status through regulatory mechanisms and population-specific effects rather than exerting uniform effects on baseline serum vitamin D concentrations. Taken together, available evidence indicates that VDR polymorphisms function as modest, context-dependent modifiers of vitamin D signaling rather than as strong or universal predictors of vitamin D status or disease risk.

#### Vitamin D binding protein polymorphisms

Variants in the *GC* gene affect vitamin D-binding protein and influence vitamin D transport and bioavailability. SNPs such as rs2282679, rs4588, and rs7041 show strong associations with serum vitamin D concentrations (15). Across multiple independent studies, these GC variants have been associated with decreased circulating vitamin D levels (3–5, 10, 15, 16). Specifically, these SNPs have been linked to lower circulating 25-hydroxyvitamin D concentrations, reduced vitamin D–binding protein levels, and higher odds of vitamin D deficiency (4). These variants have been reported to reduce circulating vitamin D levels by approximately 10-35%, although effect sizes vary substantially across studies and populations (4). Some effects vary markedly by ancestry, particularly for rs7041, as GC allele frequencies and vitamin D-binding protein isoforms differ across populations, leading to ancestry-specific associations with circulating vitamin D levels and deficiency risk (11). In contrast, evidence for other *GC* variants is less consistent, with some studies reporting neutral or even positive associations with vitamin D levels. In addition, some variants appear to modify disease risk independently of vitamin D status; for example, the rs7041-G allele has been linked to a reduced nonalcoholic fatty liver disease risk (5). Overall, *GC* polymorphisms represent important genetic determinants of interindividual variability in vitamin D metabolism and deficiency risk. Importantly, several studies report neutral or non-significant associations for certain GC variants, emphasizing that not all polymorphisms consistently influence vitamin D status. The magnitude and direction of GC variant effects vary across populations, reflecting differences in allele frequencies, vitamin D-binding protein isoforms, and environmental context.

### Role of CYP2R1, CYP27B1, and CYP24A1 polymorphisms in vitamin D metabolism

Genetic variation in key enzymes of the vitamin D metabolic pathway plays an important role in influencing circulating vitamin D levels and biological activity. *CYP2R1*, which encodes the primary hepatic 25-hydroxylase, has been consistently identified as a major determinant of serum 25-hydroxyvitamin D concentrations, with several studies reporting that risk alleles reduce the efficiency of vitamin D conversion and are associated with lower circulating 25(OH)D levels (2, 4, 17). In contrast, polymorphisms in *CYP27B1*, the enzyme responsible for converting 25(OH)D to its active hormonal form, 1,25-dihydroxyvitamin D, do not consistently affect serum 25(OH)D concentrations but instead influence active vitamin D production and downstream immune and metabolic signaling (5, 16, 18) This distinction highlights that normal circulating 25(OH)D levels may coexist with impaired vitamin D signaling at the tissue level. Variants in *CYP24A1*, which encodes the vitamin D-inactivating 24-hydroxylase, have been associated with enhanced vitamin D degradation, thereby reducing vitamin D availability and contributing to functional vitamin D deficiency, even when circulating 25(OH)D levels appear adequate (5, 14, 19). These findings highlight that genetic variation across vitamin D activation and degradation pathways contributes to interindividual differences in vitamin D status and responsiveness. Table 1 shows all key genes involved in vitamin D metabolism.

**Table 1:** Key genes involved in vitamin D metabolism and their functional polymorphisms.

Gene	Function in vitamin D pathway	Common polymorphisms (SNPs)	Functional consequences	Associated health outcomes	*Evidence strength/replication	Study type (key evidence)
VDR	Nuclear receptor, mediates genomic actions of calcitriol	FokI, BsmI, ApaI, TaqI, Cdx2	Alters receptor function, transcriptional activity, and expression levels	Bone density, cancer risk, immune response	Mixed; many meta-analyses, heterogeneity/common null findings	Candidate-gene studies; meta-analyses (65, 66)
GC	Encodes vitamin D–binding protein (DBP) for transport	rs2282679, rs7041, rs4588	Modifies circulating 25(OH)D levels, transport efficiency	Liver disease, infectious susceptibility	Strong for 25(OH)D; replicated GWAS	GWAS; replication cohorts (67, 68)
CYP2R1	Hepatic 25-hydroxylation (first activation step)	rs10741657, rs2060793	Affects conversion efficiency to 25(OH)D	Serum vitamin D variability, diabetes risk	Strong for 25(OH)D; replicated GWAS	GWAS; replication cohorts (67, 68)
CYP27B1	Renal 1α-hydroxylase (activates 25(OH)D to 1,25(OH)₂D)	rs10877012, rs4646536	Influences production of active hormone form	Autoimmunity, metabolic traits	Limited-moderate; not a top consistent 25(OH)D GWAS locus	Case–control studies; autoimmune-focused genetics (69)
CYP24A1	Inactivates 1,25(OH)₂D via 24-hydroxylation	rs6013897	Alters vitamin D breakdown rates	Cancer, cardiovascular, bone metabolism	Strong biologic validity; moderate–strong GWAS for 25(OH)D	GWAS; Mendelian genetics; clinical/mechanistic studies (67, 70)

*Evidence strength was determined by the consistency of findings across independent cohorts, replication in genome-wide analyses where applicable, and alignment with established biological plausibility. Classifications of evidence were defined as follows: strong, indicating replicated genome-wide association findings or consistent evidence across multiple cohorts; moderate, reflecting mixed replication or support limited to specific disease contexts; and limited, indicating evidence derived from single populations or predominantly mechanistic studies.

### Epigenetic regulation of vitamin D

A core concept in nutri-epigenetics is the two-way interaction between vitamin D and epigenetic mechanisms. However, most human evidence linking vitamin D to epigenetic modifications is derived from cross-sectional and association studies and should not be interpreted as establishing causal or functional regulatory relationships. Epigenetic modifications can regulate genes involved in vitamin D metabolism, while vitamin D itself can influence epigenetic states across the genome. Several key vitamin D–related genes, including *VDR*, *CYP2R1*, *CYP27B1*, and *CYP24A1*, contain CpG-rich promoter regions that are susceptible to DNA methylation–mediated regulation, as supported by human epigenetic studies (20). Hypermethylation of vitamin D-related gene promoters has been associated with reduced gene expression in human tissues, although these observations are largely correlational and may reflect downstream effects of disease state, inflammation, or environmental exposures rather than direct regulatory mechanisms (18). Moreover, epigenetic marks are often cell-type specific, dynamic, and potentially reversible, which limits their interpretation as stable biomarkers of vitamin D responsiveness. Such epigenetic modifications may therefore impair vitamin D signaling even when serum 25-hydroxyvitamin D concentrations fall within conventionally adequate ranges. Beyond DNA methylation, Vitamin D has been shown, primarily in experimental and cell-based models, to be associated with changes in chromatin structure through histone modifications (21). VDR interacts with histone-modifying enzymes, such as acetyltransferases and deacetylases, to influence chromatin accessibility. When activated, VDR recruits co-activator complexes that modify histones at target genomic sites, thereby shaping transcriptional outcomes (22). Studies of *CYP24A1* show that vitamin D signaling triggers dynamic histone changes during gene activation (22). VDR-mediated histone modifications operate across multiple regulatory regions and contribute to broader cellular processes, including cell cycle control (23) Epigenetic alterations in vitamin D–related genes have been observed in several human disease contexts, including metabolic and reproductive disorders, where they are associated with altered vitamin D responsiveness (18). Collectively, vitamin D signaling has been shown to influence epigenomic and transcriptional regulation across multiple human tissues, particularly in immune and metabolic systems (19, 20). While these epigenetic associations provide important mechanistic hypotheses, their functional relevance *in vivo* and contribution to interindividual differences in vitamin D status remain incompletely defined.

In contrast to fixed genetic variation, epigenetic mechanisms provide a flexible regulatory layer through which environmental exposures such as sunlight availability, dietary intake, inflammatory burden, metabolic status, and life stage may shape vitamin D metabolism and signaling over time. Human epigenome-wide association studies conducted in peripheral blood have reported associations between circulating 25-hydroxyvitamin D concentrations and differential DNA methylation at multiple genomic loci, including regions related to immune regulation and metabolic pathways, with evidence that these associations may vary by season and adiposity (20). Rather than acting as primary determinants of vitamin D status, such epigenetic differences may influence the efficiency of downstream vitamin D signaling and gene regulation, providing a plausible explanation for why individuals with comparable serum 25-hydroxyvitamin D concentrations can nevertheless exhibit heterogeneous biological responses. This interpretation is supported by integrative reviews demonstrating bidirectional interactions between vitamin D signaling and the epigenome, particularly through VDR-dependent chromatin remodeling and transcriptional regulation in immune and metabolic contexts, although the majority of human evidence remains associative (2, 19).

Epigenetic regulation has also been proposed as a potential mechanism through which early-life vitamin D exposure may exert longer-term effects on immune and metabolic function. Observational studies in humans indicate that maternal circulating 25-hydroxyvitamin D concentrations during pregnancy are associated with persistent DNA methylation differences in offspring, including loci involved in immune development and inflammatory regulation, raising the possibility of vitamin D-related epigenetic programming across the life course while acknowledging that causal inference remains limited.

Although epigenetic regulation is increasingly recognized as a biologically plausible contributor to interindividual variability in vitamin D signaling, several methodological and interpretive challenges limit its current explanatory scope in human populations. Epigenetic marks are dynamic, highly tissue specific, and strongly influenced by disease state and environmental context, and most available studies rely on peripheral blood as a surrogate tissue that may not accurately reflect regulatory processes occurring in classical vitamin D target tissues such as bone, intestine, or kidney (1, 21). In addition, the predominance of cross-sectional study designs complicates causal inference, making it difficult to determine whether observed epigenetic differences represent upstream regulators of vitamin D signaling or downstream consequences of vitamin D deficiency, inflammation, or chronic disease. Consequently, while epigenetic mechanisms clearly represent an important regulatory layer within vitamin D biology and provide valuable mechanistic insight into gene–environment interactions, their functional relevance for guiding individualized vitamin D interventions remains insufficiently defined. Addressing these limitations will require longitudinal and interventional studies that integrate epigenomic data with genetic, transcriptomic, and phenotypic measures to clarify the temporal dynamics and biological significance of vitamin D-associated epigenetic variation (2). Table 2 summarizes all epigenetic mechanisms that regulate vitamin D genes.

**Table 2:** Epigenetic mechanisms regulating vitamin D pathway genes.

Gene	Epigenetic modification	Effect on expression	Biological/clinical implication	*Level of evidence	Study type (key evidence)
VDR	Promoter methylation; histone acetylation	Downregulation or activation of VDR signaling	Disease-context epigenetic patterns (e.g., colorectal cancer) and immune-response regulation	Moderate (human associative; functional linkage in primary studies)	Human case–control methylation + expression; human immunology/epigenetic regulation (71, 72)
CYP24A1	Histone modifications (e.g., H3 acetylation)	Enhanced or silenced gene activity	Strong biological plausibility for epigenetic control of vitamin D catabolism; strongest evidence is mechanistic (often cancer cell/tissue contexts)	Strong mechanistic; limited general-population clinical validation	Mechanistic promoter methylation/HDAC studies; chromatin/histone work (22, 73)
CYP2R1	CpG methylation in promoter region	Reduced enzyme transcription	Observational/clinical relevance mainly for variability in 25(OH)D (particularly response to supplementation), not yet causal	Moderate (human associative; replication mixed)	Human supplementation/response methylation study; disease-context methylation study; negative/weak-correlation cohort evidence (74, 75)

*Evidence strength was determined by the consistency of findings across independent cohorts, replication in genome-wide analyses where applicable, and alignment with established biological plausibility. Classifications of evidence were defined as follows: strong, indicating replicated genome-wide association findings or consistent evidence across multiple cohorts; moderate, reflecting mixed replication or support limited to specific disease contexts.

### Molecular actions of vitamin D

#### VDR signaling

Vitamin D affects cellular function through several interconnected mechanisms. In the classical genomic pathway, calcitriol binds to the vitamin D receptor (VDR), which then binds to vitamin D response elements in gene promoters to regulate transcription (14). Beyond this, calcitriol can has been shown to produce rapid non-genomic effects by activating intracellular kinase signaling pathways (24). Vitamin D also supports cellular protection by increasing the expression of antioxidant enzymes through VDR-dependent processes (25). Certain progenitor cells that express VDR exhibit improved activity and function when exposed to vitamin D, highlighting its broader role in tissue regulation (26). Interactions between VDR and other nuclear receptors add another layer of complexity, as these interactions can alters receptor function and influence gene regulation (27).

#### Gene regulation

VDR binding across the genome helps explain the broad physiological roles of vitamin D. By attaching to regulatory regions of many target genes, VDR influences key pathways involved in immunity, metabolism, and cellular differentiation (28). Research also shows that VDR expression differs among tissues and species, particularly within blood cells, indicating that vitamin D may have specialized functions in different physiological contexts (29). In addition, vitamin D-metabolizing enzymes are expressed at key tissue interfaces, further emphasizing the hormone’s role in maintaining systemic homeostasis (30). Taken together, these findings illustrate how vitamin D and VDR interact with the genome to regulate diverse biological processes and contribute to health and disease.

#### Omics technologies in vitamin D and nutrigenomics research

Modern nutrigenomic research on vitamin D relies heavily on genomic approaches to identify genetic determinants of vitamin D status at the population level. GWAS have been influential in identifying multiple loci associated with circulating 25-hydroxyvitamin D concentrations; however, these variants individually exert modest effects and collectively explain only a limited proportion of population-level variability. These studies have expanded earlier candidate-gene research and indicate that vitamin D status is a polygenic trait influenced by numerous variants with modest individual effects (3). More recent GWAS efforts continue to validate known associations and identify previously unrecognized variants, thereby improving understanding of interindividual variability in vitamin D levels across diverse populations (31).

Beyond genomic association studies, epigenomic and transcriptional tools have provided critical insight into how vitamin D regulates gene expression and cellular function. Chromatin immunoprecipitation followed by sequencing (ChIP-seq) has been widely used to map vitamin D receptor (VDR) binding sites across the genome, revealing thousands of vitamin D-responsive regulatory regions, particularly in immune and metabolic tissues (2, 32). Epigenetic analyses, including DNA methylation and histone modification profiling, have further shown that vitamin D signaling both influences and is influenced by the epigenetic landscape of target genes (19). Transcriptomic approaches such as RNA sequencing have complemented these findings by identifying gene expression changes induced by vitamin D exposure, helping to define VDR-regulated gene networks (2). Increasingly, integrative multi-omics and bioinformatics approaches are being applied to combine genomic, epigenomic, and transcriptomic data, research efforts toward the development of precision nutrition strategies tailored to individual vitamin D metabolism and responsiveness (33). Table 3 summarising all Omics tools related to vitamin D.

**Table 3:** Omics tools used in vitamin D nutrigenomics research.*

Omics level	Method/tool	What it detects	Relevance to vitamin D research	Study type (key evidence)
Genomics	GWAS	Common SNPs	Identifies variants affecting vitamin D levels	Genome-wide association study (18)
Epigenomics	DNA methylation arrays	CpG methylation	Defines epigenetic regulation of vitamin D genes	Case–control epigenetic study (18)
Transcriptomics	RNA-seq	Gene expression patterns	Identifies VDR target gene networks	Transcriptomic nutrigenomics study (33)
Chromatin mapping	ChIP-seq	VDR binding sites	Maps vitamin D response elements across genome	Chromatin immunoprecipitation mapping study (32)

*These approaches differ in inferential strength, with GWAS providing population-level associations, epigenomic tools identifying regulatory correlations, and chromatin mapping and transcriptomics offering mechanistic insight.

To synthesize these genomic, epigenomic, and molecular findings into a unified analytical framework that guides interpretation of downstream disease associations, we propose the integrative model with three tires summarized in Box 1.

BOX 1 **Hierarchical determinants of circulating vitamin D concentrations and functional response variability.**

Tier 1-Determinants of circulating vitamin D status (strongest population-level evidence): replicated genome-wide association studies consistently identify genes involved in vitamin D synthesis, metabolism, and transport such as *GC*, *CYP2R1*, *DHCR7/NADSYN1*, and *CYP24A1* as key contributors to interindividual variation in circulating 25-hydroxyvitamin D concentrations, although these variants collectively explain only a modest proportion of the total variance.

Tier 2-Modifiers of tissue-level signaling and responsiveness: genetic variation in the vitamin D receptor (*VDR*) and vitamin D-metabolizing enzymes, including *CYP27B1* and *CYP24A1*, appears to influence cellular sensitivity to vitamin D and downstream gene regulation rather than baseline circulating vitamin D concentrations. These variants may therefore contribute to heterogeneity in biological responses across different tissues and disease contexts.

Tier 3-Epigenetic modulation and context dependence: epigenetic processes, including DNA methylation and histone modifications, add a dynamic regulatory layer that modulates vitamin D-responsive transcriptional programs. These mechanisms may help explain why individuals with similar circulating vitamin D concentrations can exhibit markedly different biological responses.

### Health outcomes in relation to vitamin D genetics

#### Cancer

Vitamin D has been implicated in cancer prevention through several biological mechanisms. Its active form has been shown to support normal cell growth, promote programmed cell death, and slow the uncontrolled division of cancer cells (34). The vitamin D receptor (VDR) also regulates many genes involved in cancer development and tumor-related signaling pathways (35). In breast cancer cells, calcitriol has been shown to increase the production of antimicrobial peptides, although the effect depends on the cell type (36).

Genetic differences in vitamin D-related genes have been associated with variation in cancer risk, likely through modulation of vitamin D signaling pathways rather than direct effects on circulating vitamin D concentrations or strong population-level causal effects (37).

Most reported associations in this area are derived from observational or genetic association studies and should be interpreted cautiously in the absence of consistent replication or causal inference. For example, some VDR gene variants are linked to improved survival in liver cancer (38), and Higher vitamin D levels have been associated with a reduced risk of colorectal cancer in observational and genetic association studies (39). Other genetic variants in the vitamin D pathway have also been associated with prostate cancer risk, with patterns varying among different ancestry groups (40). However, evidence from large randomized trials has not demonstrated a substantial reduction in overall cancer incidence with vitamin D supplementation, suggesting that reported associations may not reflect strong population-level causal effects (41).

Vitamin D influences cancer-related pathways in part by regulating gene activity in cells. VDR has been shown to help maintain epithelial structure, regulate cell growth, and influence tumor progression (14). In estrogen receptor–negative breast cancer, calcitriol can even activate estrogen receptor genes, which may help restore sensitivity to hormone-based treatments (42). Additionally, vitamin D can has been shown to reduce the activity of hypoxia-inducible factors, which are commonly upregulated in low-oxygen tumor environments, helping to limit tumor-promoting gene expression (24).

#### Immune mediation

Vitamin D plays an essential role in regulating immune function, primarily through activation of the vitamin D receptor (VDR) in various immune cells (16). Genetic variations in the VDR gene have been linked to differences in immune responsiveness and susceptibility to autoimmune diseases, although most evidence is derived from observational and candidate-gene association studies (16). Most reported associations in this area are derived from observational or genetic association studies and should be interpreted cautiously in the absence of consistent replication or causal inference. Evidence from large randomized trials indicates that vitamin D supplementation can reduce the risk of acute respiratory tract infections, with benefits that are heterogeneous and strongly dependent on dosing regimen and baseline vitamin D status, limiting generalization to broader immune-related outcomes or populations (43). Genome-wide studies further show that VDR binds to thousands of immune related genes, indicating that vitamin D influences many pathways involved in immune cell differentiation and activation (32). Epigenetic mechanisms add another layer of regulation. Vitamin D deficiency has been associated with disrupted immune balance through alterations in DNA methylation patterns in immune genes (18). The active form of vitamin D, calcitriol, also shapes T-helper cell differentiation by modulating key transcription factors (44) and influences how dendritic cells control immune responses (45). Genetic variants in vitamin D-related genes also contribute to susceptibility to infections and other immune-mediated conditions although reported effect sizes are generally modest and context-dependent. For example, HIV infected infants with both low vitamin D levels and specific VDR polymorphisms showed a substantially higher risk of developing tuberculosis (16) VDR variants have also been associated with cervical cancer risk, a finding often interpreted in the context of altered immune responses to persistent viral infection (46) and several other immune-related diseases (47).

#### Metabolic disorders

Genetic variation within the vitamin D metabolic pathway reliably influences circulating 25-hydroxyvitamin D [25(OH)D] concentrations, with the most consistently replicated loci across populations identified in or near GC, CYP2R1, DHCR7/NADSYN1, and CYP24A1, helping explain inter-individual differences in long-term vitamin D status (3, 48, 49). However, evidence that these vitamin-D-lowering variants translate into materially higher risk of metabolic disorders is less consistent. Several single-nucleotide polymorphisms (SNPs) have been associated with differences in vitamin D status and glucose-related traits, which may contribute to variability in type 2 diabetes susceptibility (3, 11), particularly in specific populations or physiological contexts. Early genetic association work on VDR polymorphisms reported links with metabolic traits, but results have been heterogeneous across populations and study designs, and overall effect sizes are small and inconsistently replicated in larger samples. Most reported associations in this area are derived from observational or genetic association studies and should be interpreted cautiously in the absence of consistent replication or causal evidence. Mendelian randomization (MR) studies aimed at clarifying causality yield mixed results for metabolic disease outcomes: systematic reviews of MR evidence on vitamin D and type 2 diabetes concluded that genetically predicted variation in 25(OH)D is not consistently causally linked to T2D risk across studies (50), and two-sample MR analyses in younger populations also found no significant causal effect of 25(OH)D levels on youth-onset T2D risk (51). In addition to glucose metabolism, variants in the vitamin D receptor have been examined in relation to metabolic syndrome and related traits, but evidence remains variable and modest, and standardized causal MR analyses of 25(OH)D and metabolic syndrome have not supported a direct causal role (52), although modest or context-dependent effects, including those related to vitamin D signaling efficiency rather than circulating 25(OH)D concentrations, cannot be excluded.

#### Bone health

Genetic differences in the VDR gene play an important role in bone mineral density and osteoporosis risk. Among the most studied variants, the BsmI and ApaI polymorphisms have shown consistent associations with reduced bone density and higher osteoporosis prevalence (53). Other VDR variants have similarly been linked to skeletal outcomes, including fracture susceptibility and disease progression (13). Although many polymorphisms occur in the 3′ untranslated region and may act as markers rather than direct functional drivers, their clinical relevance remains significant. Genetic background can influence how individuals respond to vitamin D intake, suggesting that individuals with certain VDR genotypes may require tailored supplementation strategies to maintain adequate bone health (54). In contrast to several non-skeletal outcomes, skeletal endpoints remain the area where vitamin D biology shows the greatest consistency; however, evidence from randomized trials and meta-analyses indicates that benefits are largely confined to individuals with vitamin D-deficiency and are most evident when vitamin D is combined with calcium rather than administered alone (55).

#### Neurological and cardiovascular health

Vitamin D’s active form plays several protective roles in the nervous system, including reducing oxidative stress and dampening neuroinflammatory processes (56). Variations in the VDR gene have been linked to differences in vitamin D utilization within neural tissues, which may help explain susceptibility to certain neurodegenerative disorders (56). Genetic variants in vitamin D metabolism genes have been associated with clinical features of conditions such as Alzheimer’s and Parkinson’s disease, primarily based on candidate-gene and observational studies, with modest and population-specific effects (57). Vitamin D influences cardiovascular function through multiple mechanisms (58). Calcitriol has been shown to support vascular health by modulating pathways related to oxidative stress and endothelial activity (58). Genetic determinants of vitamin D status, particularly variants affecting VDR and enzymes in the metabolic pathway, have been investigated in relation to cardiovascular risk, with associations generally modest and dependent on specific SNPs and ancestry (7, 59). Specific VDR polymorphisms, including BsmI and FokI, have been associated with essential hypertension in meta-analyses, although effect sizes are small and findings vary across populations (59, 60). Additional variants within the vitamin D pathway have been linked to a range of metabolic traits, including alterations in lipid metabolism, insulin sensitivity, and overall metabolic risk (12, 61). These findings highlight the broad systemic effects of vitamin D genetic, while emphasizing the importance of cautious interpretation and the role of nutrigenomics in explaining inter-individual variability. Table 4 shows health conditions related to different genes.

**Table 4:** Health conditions associated with vitamin D-related genetic variants.

Health condition	Gene/SNP	Mechanistic link	*Consistency of evidence	Study type (key evidence)
Type 2 diabetes	VDR (FokI, BsmI, ApaI, TaqI)	Regulates calcium absorption and bone remodeling	Moderate; model- and population-dependent	Meta-analysis (76)
Gestational diabetes	VDR rs7975232 (ApaI); (others assessed: rs1544410, rs2228570, rs731236)	Influences insulin secretion and sensitivity via VDR signaling	Limited–moderate; signal strongest for rs7975232; heterogeneity for others	Systematic review + meta-analysis (74)
Osteoporosis	VDR (FokI, TaqI; BsmI, ApaI)	Modulates glucose metabolism during pregnancy	Moderate–strong overall, but ancestry- and model-dependent	Meta-analyses (11, 77)
Autoimmune disease	VDR SNPs in AITD and SLE	Modulates T-cell differentiation and cytokine signaling	Moderate; varies by autoimmune phenotype and ancestry	Meta-analyses/pooled analysis (78, 79)
Breast cancer	VDR (Bsm1, Apa1, Fok1, Poly(A); also assessed: Cdx2, Bgl1, Taq1)	Influences cell proliferation and differentiation pathways	Limited–moderate; mixed by SNP	Systematic review + meta-analysis (65)
NAFLD	GC/DBP variants; VDR rs2228570; CYP24A1, CYP27B1 variants	Vitamin D transport and catabolism may influence hepatic metabolism	Limited–moderate; population- and design-dependent	Cohort + case–control (5, 80)
Hypertension	VDR BsmI; VDR FokI	VDR signaling implicated in vascular and endocrine pathways	Moderate; SNP- and ancestry-dependent	Meta-analyses (59, 81)
Alzheimer’s disease/MCI	VDR (ApaI, BsmI, TaqI)	Proposed effects on vitamin D utilization and neuroprotection	Limited–moderate; population discrepancies noted	Systematic review + meta-analysis (45)

*Evidence strength was determined by the consistency of findings across independent cohorts, replication in genome-wide analyses where applicable, and alignment with established biological plausibility. Classifications of evidence were defined as follows: strong, indicating replicated genome-wide association findings or consistent evidence across multiple cohorts; moderate, reflecting mixed replication or support limited to specific disease contexts; and limited, indicating evidence derived from single populations or predominantly mechanistic studies.

### Precision nutrition and personalized supplementation

While nutrigenomic research has improved understanding of interindividual variability in vitamin D metabolism, current genetic and epigenetic evidence remains insufficient to support routine genotype-guided vitamin D supplementation in clinical practice.

#### Gene–environment interactions

Vitamin D supplementation does not produce uniform results across individuals because genetic differences influence vitamin D metabolism and physiological responsiveness. Several VDR polymorphisms affect how effectively serum vitamin D levels rise after supplementation, shaping both metabolic and functional outcomes (15). Variants in genes encoding vitamin D-binding protein also contribute to this variability, emphasizing the important role of genetics in determining overall vitamin D status (15). Environmental factors such as sunlight exposure, dietary habits, and lifestyle interact with these genetic variations, creating unique vitamin D profiles across individuals and populations (3). However, these interactions primarily inform mechanistic understanding and risk stratification hypotheses rather than validated clinical decision-making tools.

#### Personalized nutrition approaches

Precision nutrition frameworks propose structured approaches for using genetic information to explore individualized dietary recommendations, although their clinical utility for vitamin D supplementation remains under active investigation. These approaches progress from general population-based guidelines to more targeted, genotype informed interventions, and they are increasingly being explored in the context of vitamin D (33). In clinical research settings, genetic screening may help identify individuals at higher risk of vitamin D deficiency, although its clinical utility remains under investigation (3). Polygenic risk scores that incorporate several vitamin D-related variants may improve prediction accuracy; however, their performance is highly population dependent and currently limited by ancestry bias in available genetic datasets (3). In addition, the predictive performance of vitamin D-related polygenic risk scores is constrained by modest individual effect sizes and limited cross-population portability. Responses to cholecalciferol supplementation, including changes in both circulating 25-hydroxyvitamin D levels and downstream biological responses, have been associated with common vitamin D-related SNPs (10). Combining serum monitoring with genetic data may, in the future, enhance individualized vitamin D management, pending validation in large, prospective clinical trials (3). At present, direct measurement of serum 25-hydroxyvitamin D remains the most reliable and clinically actionable indicator of vitamin D status, outperforming genetic stratification for routine assessment and management.

#### Individual responsiveness and personalized dosing

**Figure 4:**
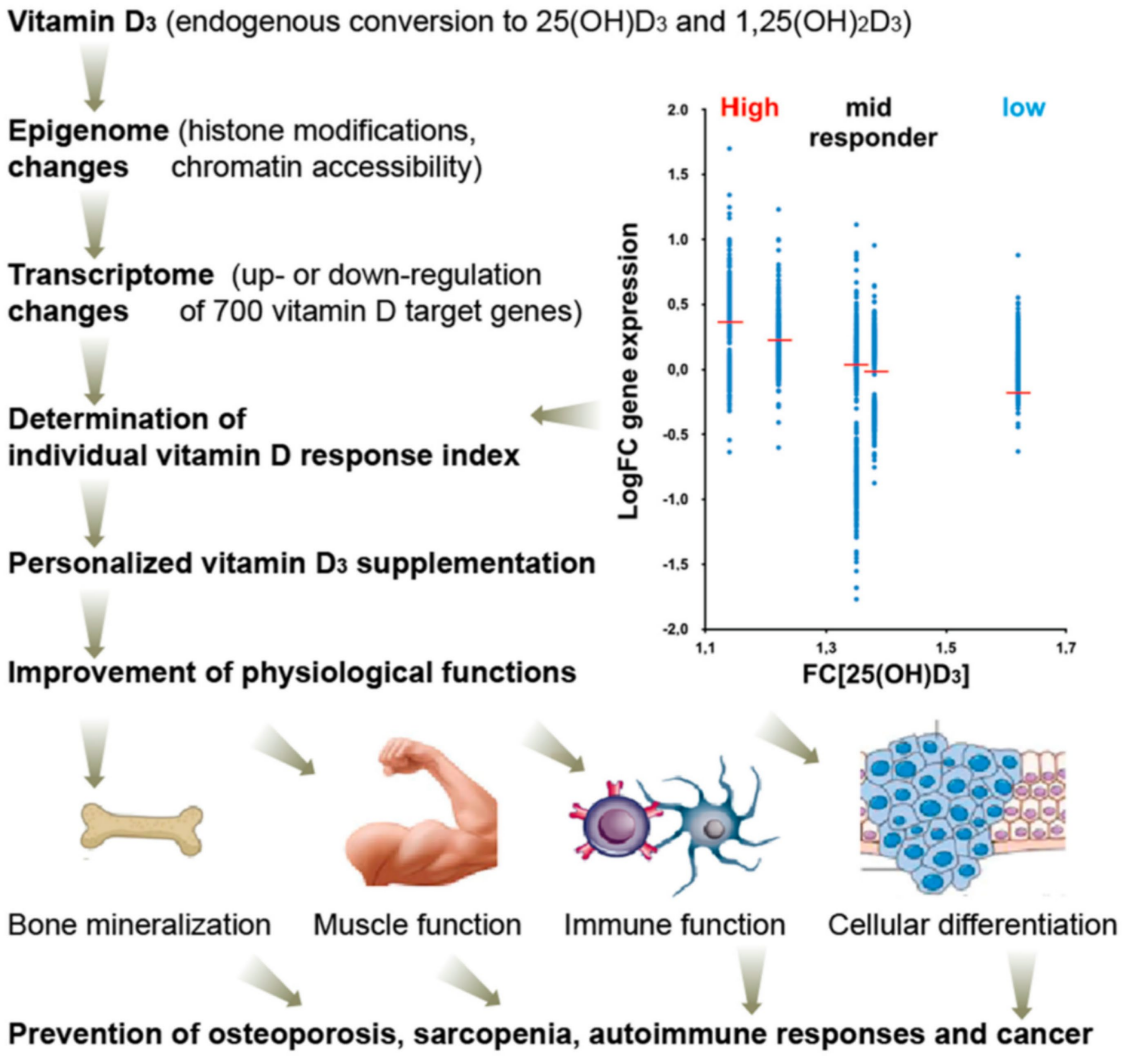
Individual variability in vitamin D response. Adapted from Carlberg (2), licensed under CC BY 4.0. Vitamin D₃ induces epigenomic modifications and transcriptomic changes across hundreds of target genes. Variation in these responses allows classification into high, mid, and low responders, illustrating variability in vitamin D response phenotypes that may inform future personalized supplementation research.

Several studies indicate that VDR polymorphisms primarily affect downstream biological responsiveness to vitamin D supplementation, with more variable effects on changes in serum 25-hydroxyvitamin D levels, and with some studies reporting relatively stronger responses in specific genotypic groups (8). Meta-analyses have identified genotype-stratified associations, suggesting that individual responses to vitamin D supplementation may be influenced by genetic factors. These gene–nutrient interactions have been documented across multiple clinical and observational studies, expanding mechanistic understanding of how vitamin D status may relate to health outcomes (62) This concept is illustrated in Figure 4, which shows how vitamin D triggers epigenomic and transcriptomic changes that vary between individuals, giving rise to high-, mid-, and low-response phenotypes that underpin personalized supplementation strategies. At present, genotype-guided vitamin D supplementation remains investigational and is not yet supported by standardized clinical guidelines.

### Strengths and limitations

A major strength of this narrative review is its comprehensive and integrative nutrigenomic perspective, which brings together genetic, epigenetic, molecular, and nutrigenomic evidence across the full vitamin D metabolic pathway. Rather than focusing on a single gene or isolated polymorphism, this review synthesizes findings related to vitamin D synthesis (CYP2R1, CYP27B1), transport (GC), metabolism (CYP24A1), and receptor signaling (VDR), thereby providing a unified framework for understanding interindividual variability in vitamin D metabolism and biological responsiveness. The inclusion of evidence from diverse study designs-including genome-wide association studies, candidate-gene and clinical association studies, epigenetic investigations, and mechanistic molecular research-strengthens the biological plausibility of the observed associations and enhances the relevance of this work for precision nutrition. In addition, the integration of omics technologies and gene–environment interactions highlight how genetic information may inform more individualized vitamin D supplementation strategies in the future. Differences in evidentiary strength across GWAS, candidate-gene, epigenetic, and mechanistic studies were considered qualitatively but could not be formally weighted within a narrative review framework.

Several limitations should nonetheless be acknowledged. This review is narrative in nature and does not follow a formal systematic review or meta-analytic framework; therefore, heterogeneity across populations, study designs, and outcome measures could not be quantitatively assessed. Formal risk-of-bias instruments and publication-bias assessments were not applied, as these approaches are not methodologically transferable across genome-wide association studies, epigenetic association studies, and mechanistic experiments. Instead, study credibility was evaluated qualitatively, with emphasis placed on replication consistency, ancestry representation, and convergence of findings across complementary study designs. Much of the available evidence is derived from observational and association studies, which limits the ability to infer causality between genetic variants and vitamin D-related health outcomes. Moreover, the majority of genetic studies focus on common single-nucleotide polymorphisms, whereas the contributions of rare variants, gene–gene interactions, and structural genetic variation remain underexplored, and functional characterization of several widely studied noncoding variants is still incomplete. Finally, differences in ancestry representation, allele frequencies, linkage disequilibrium patterns, vitamin D assessment methods, epigenetic platforms, and supplementation protocols across studies may affect the generalizability and reproducibility of findings. These limitations underscore the need for large, well-designed, multi-ethnic clinical trials and integrative multi-omics approaches to support the translation of nutrigenomic insights into clinical practice.

### Future research directions

Advancing the integration of nutrigenomics into clinical practice will require large, multicenter randomized controlled trials specifically designed to test genotype-guided vitamin D supplementation strategies (63). These trials must account for both genetic variation and environmental influences to more accurately predict individual vitamin D requirements (64). Further progress will depend on building predictive models using multi-omics datasets that combine genetic, metabolic, epigenetic, and environmental variables (33). Finally, research on rare genetic variants remains essential, as these may explain differences in vitamin D metabolism not accounted for by common SNPs (3).

## Conclusion

The integration of vitamin D research with nutrigenomics has greatly enhanced our understanding of why individuals differ in vitamin D status, metabolism, and biological responsiveness. Genetic variations in key components of the vitamin D pathway particularly VDR, GC, CYP2R1, CYP27B1, and CYP24A1, contribute to measurable differences in circulating vitamin D levels and tissue sensitivity. Epigenetic mechanisms further modulate the expression of vitamin D-related genes, illustrating the bidirectional relationship between vitamin D signaling and the epigenome. These insights underscore the growing relevance of precision nutrition. Incorporating genetic information into research frameworks may improve identification of individuals at greater risk of deficiency, pending validation in large, prospective clinical studies. Successful translation of these findings into practice will depend on the development of standardized interpretive frameworks and validated biomarkers that can reliably guide clinical decision-making.

Overall, the vitamin D endocrine system influences a wide range of physiological processes and interacts with a substantial portion of the human genome. As evidence accumulates, Vitamin D is increasingly studied as a model nutrient for exploring precision medicine approaches within nutrition science. Maintaining adequate vitamin D levels remains essential for population health, while acknowledging that genetic polymorphisms and environmental factors contribute to meaningful variation in individual requirements.
